# LPS preconditioning redirects TLR signaling following stroke: TRIF-IRF3 plays a seminal role in mediating tolerance to ischemic injury

**DOI:** 10.1186/1742-2094-8-140

**Published:** 2011-10-14

**Authors:** Keri B Vartanian, Susan L Stevens, Brenda J Marsh, Rebecca Williams-Karnesky, Nikola S Lessov, Mary P Stenzel-Poore

**Affiliations:** 1 Department of Molecular Microbiology & Immunology, Oregon Health & Science University, Portland, OR 97239, USA

**Keywords:** Toll-like receptors, stroke, NFκB, inflammation, preconditioning, neuroprotection

## Abstract

**Background:**

Toll-like receptor 4 (TLR4) is activated in response to cerebral ischemia leading to substantial brain damage. In contrast, mild activation of TLR4 by preconditioning with low dose exposure to lipopolysaccharide (LPS) prior to cerebral ischemia dramatically improves outcome by reprogramming the signaling response to injury. This suggests that TLR4 signaling can be altered to induce an endogenously neuroprotective phenotype. However, the TLR4 signaling events involved in this neuroprotective response are poorly understood. Here we define several molecular mediators of the primary signaling cascades induced by LPS preconditioning that give rise to the reprogrammed response to cerebral ischemia and confer the neuroprotective phenotype.

**Methods:**

C57BL6 mice were preconditioned with low dose LPS prior to transient middle cerebral artery occlusion (MCAO). Cortical tissue and blood were collected following MCAO. Microarray and qtPCR were performed to analyze gene expression associated with TLR4 signaling. EMSA and DNA binding ELISA were used to evaluate NFκB and IRF3 activity. Protein expression was determined using Western blot or ELISA. MyD88-/- and TRIF-/- mice were utilized to evaluate signaling in LPS preconditioning-induced neuroprotection.

**Results:**

Gene expression analyses revealed that LPS preconditioning resulted in a marked upregulation of anti-inflammatory/type I IFN-associated genes following ischemia while pro-inflammatory genes induced following ischemia were present but not differentially modulated by LPS. Interestingly, although expression of pro-inflammatory genes was observed, there was decreased activity of NFκB p65 and increased presence of NFκB inhibitors, including Ship1, Tollip, and p105, in LPS-preconditioned mice following stroke. In contrast, IRF3 activity was enhanced in LPS-preconditioned mice following stroke. TRIF and MyD88 deficient mice revealed that neuroprotection induced by LPS depends on TLR4 signaling via TRIF, which activates IRF3, but does not depend on MyD88 signaling.

**Conclusion:**

Our results characterize several critical mediators of the TLR4 signaling events associated with neuroprotection. LPS preconditioning redirects TLR4 signaling in response to stroke through suppression of NFκB activity, enhanced IRF3 activity, and increased anti-inflammatory/type I IFN gene expression. Interestingly, this protective phenotype does not require the suppression of pro-inflammatory mediators. Furthermore, our results highlight a critical role for TRIF-IRF3 signaling as the governing mechanism in the neuroprotective response to stroke.

## Background

Stroke is one of the leading causes of death and the leading cause of morbidity in the United States [1]. The inflammatory response to stroke substantially exacerbates ischemic damage. The acute activation of the NFκB transcription factor has been linked to the inflammatory response to stroke [2] and suppression of NFκB activity following stroke has been found to reduce damage [3]. NFκB activation can lead to the dramatic upregulation of inflammatory molecules and cytokines including TNFα, IL6, IL1β, and COX2 [2]. The source of these inflammatory molecules in the acute response to stroke appears to stem from the cells of the central nervous system (CNS), including neurons and glial cells [2]. The cells in the CNS play a particularly dominant role early in the response to ischemia because infiltrating leukocytes do not appear in substantial numbers in the brain until 24 hr following injury [4]. Stroke also induces an acute inflammatory response in the circulating blood. Inflammatory cytokine and chemokine levels, including IL6, IL1β, MCP-1 and TNFα are elevated in the circulation following stroke [5]. This suggests there is an intimate relationship between responses in the brain and blood following stroke--responses that result in increased inflammation.

Toll-like receptors (TLRs), traditionally considered innate immune receptors, signal through the adaptor proteins MyD88 and TRIF to activate NFκB and interferon regulatory factors (IRFs). It has been shown recently that TLRs become activated in response to endogenous ligands, known as damage associated molecular patterns (DAMPs), released during injury. Interestingly, animals deficient in TLR2 or TLR4 have significantly reduced infarct sizes in several models of stroke [6-11]. This suggests that TLR2 and TLR4 activation in response to ischemic injury exacerbates damage. In addition, a recent investigation in humans showed that the inflammatory responses to stroke in the blood were linked to increased TLR2 and TLR4 expression on hematopoetic cells and associated with worse outcome in stroke [12]. The detrimental effect of TLR signaling is associated with the pathways that lead to NFκB activation and pro-inflammatory responses. In contrast, TLR signaling pathways that activate IRFs can induce anti-inflammatory mediators and type I IFNs that have been associated with neuroprotection [13,14]. Thus, in TLR signaling there is a fine balance between pathways leading to injury or protection.

TLR ligands have been a major source of interest as preconditioning agents for prophylactic therapy against ischemic injury. Such therapies would target a population of patients that are at risk of ischemia in the setting of surgery [15-18]. Preconditioning with low doses of ligands for TLR2, TLR4, and TLR9 all successfully reduce infarct size in experimental models of stroke [19-21], including a recent study showing that a TLR9 ligand is neuroprotective in a nonhuman primate model of stroke [22]. Emerging evidence suggests that TLR-induced neuroprotection occurs by reprogramming the genomic response to the DAMPs, which are produced in response to ischemic injury. In this reprogrammed state, the resultant pathway activation of TLR4 signaling preferentially leads to IRF-mediated gene expression [13,14]. However, whether TLR preconditioning affects NFκB activity and pro-inflammatory signaling is unknown. As yet, a complete analysis of the characteristic TLR signaling responses to stroke following preconditioning has not been reported. The objective of this study is to utilize LPS preconditioning followed by transient middle cerebral artery occlusion (MCAO) to elucidate the reprogrammed TLR response to stroke and to determine the major pathways involved in producing the neuroprotective phenotype.

Here we show that preconditioning against ischemia using LPS leads to suppressed NFκB activity--although pro-inflammatory gene expression does not appear to be attenuated. We also demonstrate that LPS-preconditioned mice have enhanced IRF3 activity and anti-inflammatory/type I IFN gene expression in the ischemic brain. This expression pattern was recapitulated in the blood where plasma levels of pro-inflammatory cytokine proteins were comparable in LPS-preconditioned and control mice while IRF-associated proteins were enhanced in LPS preconditioned mice. To our knowledge, we provide the first evidence that protection due to LPS preconditioning stems from TRIF signaling, the cascade that is associated with IRF3 activation, and is independent of MyD88 signaling. These molecular features suggest that, following stroke, signaling is directed away from NFκB activity and towards a dominant TRIF-IRF3 response. Understanding the endogenous signaling events that promote protection against ischemic injury is integral to the identification and development of novel stroke therapeutics. In particular, the evidence presented here further highlights a key role for IRF3 activity in the protective response to stroke.

## Methods

### Animals

C57Bl/6J mice (male, 8-12 weeks) were purchased from Jackson Laboratories (West Sacramento, CA). C57Bl/6J-Ticam1^LPS2^/J (TRIF-/-) mice were also obtained from Jackson Laboratories. MyD88-/- mice were a kind gift of Dr. Shizuo Akira (Osaka University, Osaka Japan) and were bred in our facility. All mice were housed in an American Association for Laboratory Animal Care-approved facility. Procedures were conducted according to Oregon Health & Science University, Institutional Animal Care and Use Committee, and National Institutes of Health guidelines.

### LPS treatment

Mice were preconditioned with LPS (0.2 or 0.8 mg/kg, *Escherichia coli *serotype *0111:B4*; Sigma) or saline by one subcutaneous injection, unless otherwise indicated, 72 hr prior to MCAO. Each new lot of LPS was titrated for the optimal dose that confers neuroprotection. No differences were observed in the genomic responses to LPS for each dose used and route of administration (subcutaneous or intraperitoneal, data not shown).

### Middle Cerebral Artery Occlusion (MCAO)

Mice were anesthetized with isoflurane (1.5-2%) and subjected to MCAO using the monofilament suture method described previously [23]. Briefly, a silicone-coated 7-0 monofilament nylon surgical suture was threaded through the external carotid artery to the internal carotid artery to block the middle cerebral artery, and maintained intraluminally for 40 to 60 min. The suture was then removed to restore blood flow. The duration of occlusion was optimized based on the specific surgeon who performed the MCAO to yield comparable infarct sizes in the saline treated control animals (~35-40%). The selected duration of MCAO was held constant within experiments. Cerebral blood flow (CBF) was monitored throughout surgery by laser doppler flowmetry. Any mouse that did not maintain a CBF during occlusion of <25% of baseline was excluded from the study. The reduction of CBF was comparable in LPS and saline preconditioned mice in response to MCAO. Body temperature was monitored and maintained at 37°C with a thermostat-controlled heating pad. Infarct measurements were made using triphenyltetrazolium chloride (TTC) staining of 1 mm coronal brain sections.

### Tissue collection

Under deep isoflurane anesthesia, approximately ~0.5-1.0 ml of blood was collected via cardiac puncture in a heparinized syringe. Subsequently, the mice were perfused with heparinized (2 U/ml) saline followed by rapid removal of the brain. The olfactory bulbs were removed and the first 4 mm of tissue was collected beginning at the rostral end. The striatum was dissected and removed and the remaining cortex was utilized for RNA isolation or protein extraction. The collected blood was centrifuged at 5000 × g for 20 min to obtain plasma that was stored at -80°C.

### Genomic profiling of TLR associated mediators

**Figure 1:**
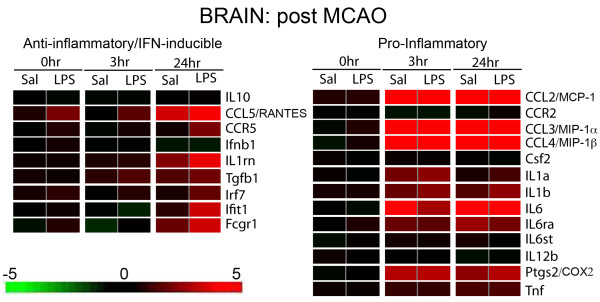
**Microarray analysis of anti-inflammatory/type I IFN and pro-inflammatory gene expression**. Microarray analysis revealed enhanced anti-inflammatory/type I IFN and comparable pro-inflammatory gene expression profiles in the brain of LPS-preconditioned (0.2 mg/kg, intraperitoneal injection) mice following 45 min MCAO. Heatmap representing level of gene expression immediately prior to (0 hr) MCAO and 3 and 24 hr post MCAO; n = 4/treatment/timepoint. Lt. Select anti-inflammatory/type I IFN genes. Rt. Select pro-inflammatory genes. Color scale from green to red represents relative decreased or increased gene expression levels, respectively.

For the genes displayed in Figure 1, the transcript expression levels were determined as previously described from our microarray experiments examining the brain cortical response to stroke and 3 different preconditioning stimuli [14]. In brief, total RNA was isolated from the ipsilateral cortex (n = 4 mice/treatment/timepoint), using the Qiagen Rneasy Lipid Mini Kit (Qiagen). Microarray assays were performed in the Affymetrix Microarray Core of the Oregon Health & Science University Gene Microarray Shared Resource. RNA samples were labeled using the NuGEN Ovation Biotin RNA Amplification and Labeling System_V1. Hybridization was performed as described in the Affymetrix technical manual (Affymetrix) with modification as recommended for the Ovation labeling protocol (NuGEN Technologies). Labeled cRNA target was quality-checked based on yield and size distribution. Quality-tested samples were hybridized to the MOE430 2.0 array. The array image was processed with Affymetrix GeneChip Operating Software (GCOS). Affymetrix CEL files were then uploaded into GeneSifter (http://www.genesifter.net) and normalized using RMA.

### RNA isolation, Reverse Transcription, and qtPCR

RNA was isolated from cortical tissue 72 hr post injection or from ipsilateral cortical tissue at 3 or 24 hr following MCAO (n ≥ 4 mice/treatment/timepoint) using a Lipid Mini RNA isolation kit (Qiagen). Reverse transcription was performed on 2 μg of RNA using Omniscript (Qiagen). Quantitative PCR was performed using Taqman Gene Expression Assays (Applied Biosystems) for each gene of interest on an ABI Prism 7700. Results were normalized to β-Actin expression and analyzed relative to their saline preconditioned counterparts. The relative quantification of the gene of interest was determined using the comparative CT method (2^-DDCt^).

### Western Blot

Protein extraction was performed as described previously [24] with some modifications. Briefly, tissue samples (n ≥ 4 mice/treatment/timepoint) were dissected from the ipsilateral cortex and lysed in a buffer containing a protease inhibitor cocktail (Roche). Protein concentrations were determined using the BCA method (Pierce-Endogen). Protein samples (50 μg) were denatured in a gel-loading buffer (Bio-Rad Laboratories) at 100°C for 5 min and then loaded onto 12% Bis-Tris polyacrylamide gels (Bio-Rad Laboratories). Following electrophoresis, proteins were transferred to polyvinylodene difluoride membranes (Bio-Rad Laboratories) and incubated with primary antibodies for Ship-1 (Santa Cruz, sc8425), Tollip (AbCam, Ab37155), p105 (Santa Cruz, sc7178), or β-Actin (Santa Cruz, sc1616R) at 4°C overnight. Membranes were then incubated with horseradish peroxidase conjugated anti-rabbit, anti-goat, or anti-mouse antibody (Santa Cruz Biotechnology) and detected by chemiluminescence (NEN Life Science Products) and exposed to Kodak film (Biomax). Images were captured using an Epson scanner and the densitometry of the gel bands, including β-Actin loading control, was analyzed using ImageJ (NIH).

### Electrophoretic Mobility Shift Assay (EMSA)

Nuclear protein extracts (n = 4 mice/treatment/timepoint) were prepared from tissue dissected from the ipsilateral cortex. Homogenized tissue was incubated in Buffer A (10 mM Hepes-KOH pH7.9, 60 mM KCl, 1 mM EDTA, 1 mM DTT, 1 mM PMSF) for 5 min on ice and centrifuged at 3000 rpm for 5 min at 4°C. The pellets were washed in Buffer B (10 mM Hepes-KOH pH7.9, 60 mM KCl, 1 mM EDTA, 0.5% NP-40, 1 mM DTT, 1 mM PMSF), resuspended in Buffer C (250 mM Tris pH7.8, 60 mM KCl, 1 mM DTT, 1 mM PMSF), and freeze-thawed 3 times in liquid nitrogen. All buffers contained a protease inhibitor cocktail (Roche). After centrifuging at 10,000 rpm for 10 min at 4°C, the supernatant was collected and stored as nuclear extract at -80°C. Nuclear protein concentrations were determined using the BCA method (Pierce-Endogen). EMSAs were performed using the Promega Gel Shift Assay System according to the manufacturer's instructions. Briefly, 15 μg of nuclear protein was incubated with ^32^P-labeled NFκB consensus oligonucleotide (Promega), either with or without unlabeled competitor oligonucleotide, unlabeled noncompetitor oligonucleotide, or anti-p65 antibody (Santa Cruz). Samples were electrophoresed on a 4% acrylamide gel, dried and exposed to phosphorimager overnight. The densitometry of the gel bands was analyzed using scanning integrated optical density software (ImageJ).

#### IRF3 Activity Assay

Nuclear protein (n ≥ 4 mice/treatment/timepoint) was isolated from fresh cortical tissue at 72 hr post injection and from ipsilateral cortices at 3 or 24 hr following MCAO using a Nuclear Extraction Kit (Active Motif, Inc.). IRF3 activity was measured using 10 μg of nuclear protein in an IRF3 activity ELISA (Active Motif, Inc), that utilizes colorimetric detection of active IRF3 bound to immobilized oligonucleotides.

### Cytokine Analysis

Cytokine/chemokine analysis for IL1β, IL1α, MIP-1α, MCP-1, RANTES, and IL10 was performed on plasma samples (n ≥ 3 mice/treatment/timepoint) using a multiplex ELISA (Quansys). An IFNβ ELISA (PBL Interferon Source) was used to measure plasma levels of IFNβ.

### Statistical Analysis

Data is represented as mean ± SEM. The n for each experiment is greater than or equal to 3, as specified in each figure. Statistical analysis was performed using GraphPad Prism5 software. Two-way ANOVA with Bonferroni Post Hoc test and Student's t-test were utilized as specified. Significance was determined as p < 0.05.

## Results

### LPS preconditioning does not affect inflammatory gene expression in the brain following stroke

**Figure 2:**
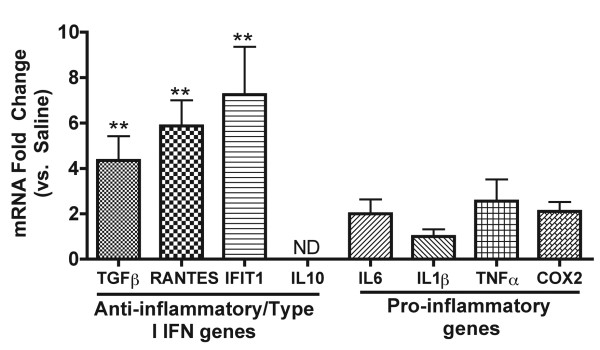
**Enhanced anti-inflammatory/type I IFN gene expression but comparable pro-inflammatory gene expression in LPS-preconditioned mice post MCAO**. Gene regulation 24 hr post MCAO measured by qtPCR reveals that anti-inflammatory/type I IFN-associated genes TGFβ, RANTES, and IFIT1 are significantly upregulated in LPS preconditioned mice compared to saline. Pro-inflammatory genes IL6, IL1β, COX2, and TNFα show similar regulation in LPS and saline preconditioned mice. These results confirm the gene microarray data. Samples from mice receiving a 45 (LPS: 0.2 mg/kg) or 60 min (LPS: 0.8 mg/kg) MCAO were combined due to comparable gene regulation (see methods). ND = not detected. Student's t-test, LPS vs. saline 24 hr post MCAO, **p < 0.01, n ≥ 4 per treatment.

We used gene microarray analysis to elucidate the pattern of inflammatory or anti-inflammatory/type I IFN gene expression in the brain following stroke. In the setting of stroke, LPS preconditioned animals exhibited regulation of a number of genes typically found downstream of TLR signaling. The inflammatory profile reveals that the gene regulation is similar at each timepoint following stroke in LPS or saline preconditioned animals (Figure 1, Rt.). There is no evidence of inflammatory gene expression present immediately prior to stroke (Figure 1, Rt. 0 hr). At 3 hr post MCAO, several inflammatory genes are upregulated including IL6, IL1β, Ptgs2/COX2, and CCL2/MCP-1 (Figure 1, Rt.) and this upregulation is sustained at the 24 hr timepoint following MCAO (Figure 1, Rt.). TNFα, which is commonly shown to be upregulated following MCAO [25,26], only shows marginal levels of upregulation in LPS or saline preconditioned mice (Figure 1, Rt.). To confirm the microarray results, a subset of selected inflammatory genes including IL6, IL1β, COX2, and TNFα, were analyzed using qtPCR. Each of these genes were upregulated following MCAO in LPS and saline preconditioned mice, but there were no significant differences based on treatment at 3 hr (data not shown) and 24 hr (Figure 2) following MCAO.

### LPS preconditioning upregulates anti-inflammatory/type I IFN gene expression in the brain following MCAO

Although pro-inflammatory gene expression was not differentially modulated in preconditioned animals, microarray results revealed that the majority of the anti-inflammatory/type I IFN genes, such as TGFβ, IL1 receptor antagonist (IL1rn), RANTES, and IRF7, were upregulated following stroke in the brains of LPS versus saline preconditioned mice (Figure 1, Lt.). IL10 gene expression was not detected at any timepoint (Figure 1, Lt.). TGFβ, IL10, RANTES, and IFIT1 were selected for qtPCR analysis. TGFβ, RANTES, and IFIT1 were significantly upregulated in the LPS-preconditioned brain compared to saline 24 hr following stroke (Figure 2). RANTES was also significantly upregulated at 3 hr following stroke in LPS-preconditioned mice compared to saline (data not shown). IL10 expression remained undetectable by qtPCR analysis (Figure 2), suggesting that IL10 mRNA is not present at these timepoints in the brain following stroke. These qtPCR results confirm the gene expression profile observed on the microarray. Taken together, these data indicate an enhanced anti-inflammatory/type I IFN gene expression profile in the brain of LPS-preconditioned animals following MCAO while the inflammatory gene expression is unaffected.

### NFκB activity is suppressed in the brain of LPS-preconditioned animals 24 hr post MCAO

**Figure 3:**
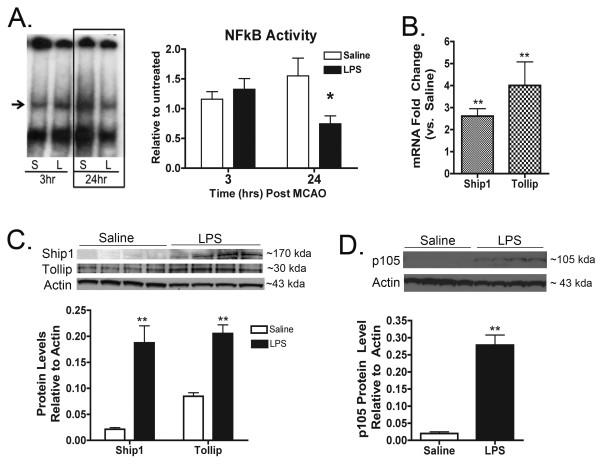
**NFκB is suppressed 24 hr post MCAO in LPS-preconditioned mice**. **(A) **Nuclear protein obtained from ipsilateral cortices was used to measure p65 activity by EMSA analysis. EMSA gel of pooled samples (n = 4) following 45 min MCAO for saline and LPS preconditioned (0.8 mg/kg) mice (Lt.). Quantification of band intensity of individual mice following MCAO (Rt.). NFκB is significantly decreased in LPS-preconditioned mice 24 hr post MCAO compared to saline. Supershift assay confirmed specificity for p65 oligos (data not shown). **(B) **Ship1 and Tollip mRNA are significantly upregulated 24 hr post 60 minute MCAO in LPS-preconditioned (0.8 mg/kg) mice compared to saline, n ≥ 4 per treatment/timepoint. **(C) **Western blot for Ship1 and Tollip and relative band quantification showing significant upregulation of Ship1 and Tollip protein 24 hr post 45 min MCAO in LPS-preconditioned mice (0.8 mg/kg), n ≥ 3 per treatment/timepoint. (**D**) Western blot and relative band quantification for p105 at 24 hr post 45 minute MCAO showing significant upregulation in LPS-preconditioned (0.8 mg/kg) mice, n ≥ 3 per treatment/timepoint. **(A) **Two-Way ANOVA, Bonferroni Post Hoc, *p < 0.05. **(B-D) **Student's t-test, LPS vs. saline, **p < 0.01.

NFκB activity is associated with damage and inflammation in the brain that occurs in response to stroke. We used EMSAs to evaluate the activity of the NFκB subunit p65 in the brain following stroke. The results indicated that LPS and saline preconditioned mice have comparable NFκB activity at 3 hr post MCAO (Figure 3A). However, at 24 hr post MCAO, LPS-preconditioned animals have significantly suppressed NFκB activity compared to saline preconditioned mice (Figure 3A).

Ship1 and Tollip are cytosolic molecules that inhibit TLR signaling, which leads to the suppression of NFκB activity. We found that Ship1 and Tollip mRNA are upregulated in the brain 72 hr post injection versus saline controls (2.06 ± 0.27 and 2.31 ± 0.35, respectively) but not at 3 hr post stroke (1.09 ± 0.10 and 1.05 ± 0.09, respectively). However, by 24 hr post MCAO, Ship1 and Tollip mRNA are significantly enhanced in the brain of LPS-preconditioned mice compared to saline controls (2.62 ± 0.84 and 4.01 ± 1.06, respectively, Figure 3B). Ship1 protein is not upregulated at 72 hr post injection (Fold change vs. saline: 1.01 ± 0.32), but becomes significantly enhanced in LPS-preconditioned mice at 3 hr (Fold change vs saline: 1.83 ± 0.13) and at 24 hr (Fold change vs. saline: 8.81 ± 1.54, Figure 3C) post MCAO. Tollip protein is not affected by LPS preconditioning at 72 hr post injection or 3 hr post MCAO (Fold change vs. saline: 1.42 ± 0.10 and 0.83 ± 0.10, respectively), but it is significantly enhanced in LPS-preconditioned mice compared to saline controls at 24 hr post MCAO (Fold change vs. saline: 2.42 ± 0.20, Figure 3C). Additionally, the p50 precursor protein p105, which inhibits NFκB activity by acting like an IκB molecule by sequestering NFκB in the cytosol [27,28], was significantly upregulated 24 hr post stroke in LPS-preconditioned mice compared to saline (Figure 3D). Thus, despite the upregulation of inflammatory genes, the activity of NFκB is suppressed in the late-phase of the neuroprotective response of LPS-preconditioned mice.

### IRF3 activity in the brain is enhanced following MCAO in LPS-preconditioned mice

**Figure 4:**
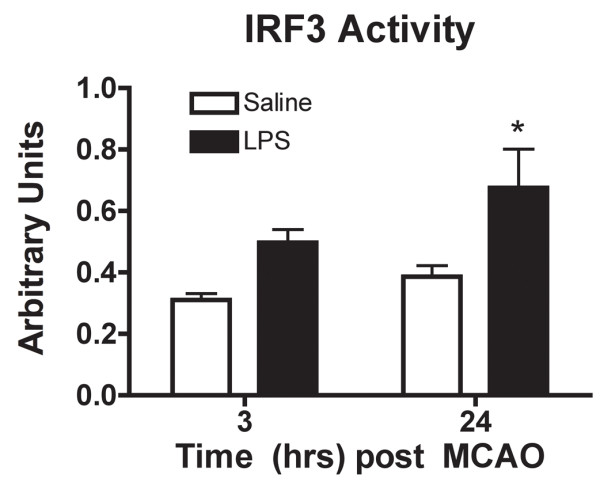
**IRF3 activity is enhanced following MCAO in LPS-preconditioned mice**. Nuclear protein obtained from ipsilateral cortex post 60 min MCAO analyzed using an IRF3 activity ELISA (Active Motif, Inc.) revealed a significant increase in IRF3 activity in LPS-preconditioned (0.8 mg/kg) mice. Two-way ANOVA, Bonferroni Post Hoc, LPS vs. saline, *p < 0.05, n ≥ 4 per treatment.

IRF3 activation downstream of TLR4 is associated with anti-inflammatory/type I IFN responses. Using an IRF3 activity ELISA, we determined that IRF3 activity is comparable immediately prior to stroke (data not shown) and subsequently enhanced in the brains of LPS-preconditioned mice following MCAO (Figure 4). The trend for increased IRF3 activity is present at 3 hr post MCAO and is significantly increased at 24 hr in LPS-preconditioned mice (Figure 4). Saline treated animals showed no evidence of increased IRF3 activity following stroke (Figure 4). This indicates that LPS preconditioning alters the response to ischemic injury by activating IRF3--a finding that is consistent with the enhanced anti-inflammatory/type I IFN gene expression.

### Blood cytokine/chemokine levels parallel the expression in the brain

**Figure 5:**
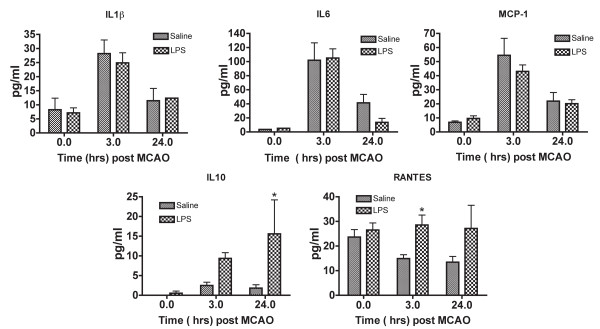
**Blood cytokine/chemokine levels show alterations in gene expression patterns comparable to the brain**. Plasma collected from saline or LPS-preconditioned (0.8 mg/kg) mice at the time of or following 60 min MCAO was examined using a multikine ELISA (Quansys). Results indicated that pro-inflammatory cytokines IL1β, IL6 and MCP-1 are similar in saline and LPS-preconditioned mice. In contrast, LPS-preconditioned mice have significantly enhanced levels of the anti-inflammatory/type I IFN-associated cytokine and chemokine IL10 and RANTES compared to saline following MCAO. Two-way ANOVA, LPS vs. saline, *p < 0.05, n ≥ 3 per treatment.

Evidence indicates that stroke alters the cytokine profile in the plasma of circulating blood [5,29]. To determine whether LPS preconditioning changes the balance of pro- and anti-inflammatory cytokines and chemokines in the plasma we examined the levels of seven molecules using ELISAs. The results indicate that the level of pro-inflammatory cytokines, such as IL6, IL1β, and MCP-1, are increased in both LPS and saline preconditioned mice (Figure 5). The pro-inflammatory cytokines MIP-1α and IL1α were not detected in the serum (data not shown). The anti-inflammatory cytokine IL10 was significantly increased only in the plasma of LPS-preconditioned mice compared to saline preconditioned mice following stroke (Figure 5). RANTES, which is a chemokine associated with IRF3 and IRF7 activity [30], was present in the blood of LPS-preconditioned mice at significantly greater levels than saline preconditioned mice (Figure 5). IFNβ was not detectable in the blood of LPS or saline preconditioned animals following stroke (data not shown). Overall, this suggests that the pro-inflammatory and anti-inflammatory/type I IFN-associated response in the blood parallels the response in the brain following stroke.

### TRIF dependent LPS preconditioning induced neuroprotection

**Figure 6:**
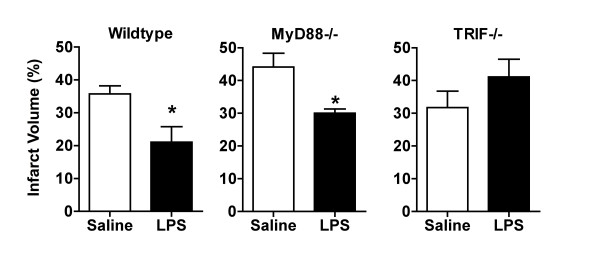
**LPS preconditioning requires TLR signaling through TRIF to promote neuroprotection**. WT, MyD88-/-, and TRIF-/- mice were preconditioned with LPS (0.8 mg/kg) 3 days prior to 40 min MCAO. MyD88-/- mice were protected by LPS preconditioning resulting in smaller infarct sizes. TRIF-/- mice did not have reduced infarct sizes, demonstrating that TRIF deficient mice are not protected by LPS preconditioning. Thus, TRIF is required for LPS preconditioning induced neuroprotection. Student's t-test, LPS vs. saline, *p < 0.05, n = 4-10 per treatment.

Evidence presented here and previously suggests that signaling following stroke is redirected towards IRF3 [13,14]. TLR4 signaling, which activates IRF3, is initiated by the adaptor molecule TRIF, while TLR4 signaling that activates NFκB is initiated by the adaptor molecule MyD88. The individual roles of these adaptor molecules in neuroprotection induced by LPS preconditioning are unknown. To test whether either of these key molecular adaptors were important in mediating the neuroprotective effects of LPS, we exposed MyD88-/- and TRIF-/- mice to LPS preconditioning (n = 4-10 mice/treatment). We found that MyD88-/- mice preconditioned with LPS had significantly reduced infarct sizes in response to MCAO compared to saline controls (Figure 6), indicating that LPS preconditioning is able to induce neuroprotection in mice lacking MyD88. In contrast, TRIF-/- mice preconditioned with LPS or saline had comparable infarct sizes (Figure 6), indicating that LPS preconditioning is not able to induce neuroprotection in mice lacking TRIF. Importantly, the TRIF adaptor is responsible for activation of IRF3, thus, our finding that TRIF is required for LPS preconditioning provides further support for a protective role of IRF3 activity in neuroprotection.

## Discussion

**Figure 7:**
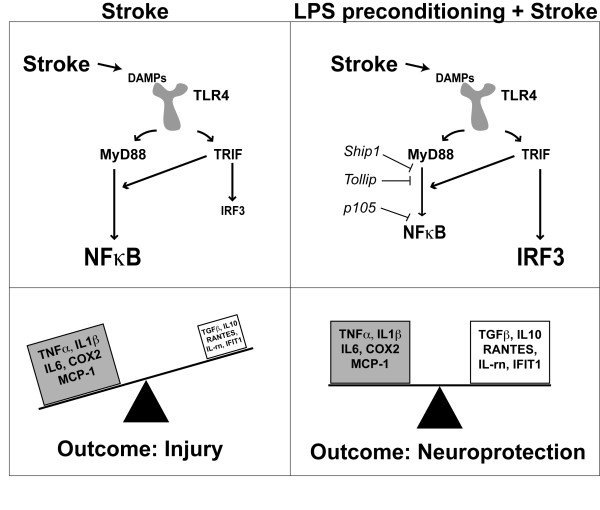
**Schematic of TLR4 signaling and gene expression following stroke**. **(Top) **TLR4 signaling cascades following stroke. In the absence of LPS preconditioning, stroke leads to NFκB activation without IRF3 activation. LPS preconditioning prior to stroke leads to robust activation of IRF3 and suppressed NFκB activity compared to stroke alone. **(Bottom) **Gene expression 24 hr post stroke. Stroke alone dramatically upregulates pro-inflammatory genes. LPS preconditioning prior to stroke dramatically upregulates anti-inflammatory/Type I IFN genes, many of which are associated with IRF3, while still maintaining a pro-inflammatory response.

Here we sought to describe the LPS-induced reprogrammed response to stroke and to determine the important signaling events involved in neuroprotection against ischemic injury. Our results demonstrated that NFκB activity was suppressed and that the cytosolic inhibitors of NFκB, Ship1, Tollip, and p105, were present 24 hr post MCAO although pro-inflammatory gene expression was unaffected (diagrammed in Figure 7). Interestingly, there is evidence that suppression of NFκB can promote protection against cerebral ischemia without influencing pro-inflammatory cytokine production [3,31]. In particular, administration of the NFκB inhibitor Tat-NEMO Binding Domain provided protection against hypoxia-ischemia in neonatal rats without affecting TNFα or IL1β production [3]. Furthermore, TLR4 deficient mice have smaller infarcts in response to MCAO, yet the production of TNFα and IL1β was unaffected [6]. This suggests that reduced ischemic injury can be achieved by suppressing NFκB activity without suppressing pro-inflammatory cytokines and that TLR4 signaling and NFκB activation is not the sole source of these pro-inflammatory cytokines in response to ischemic injury, implicating other signaling cascades and transcription factors in the inflammatory response. Thus, consistent with our result, reprogramming the TLR4 response would not alter inflammatory gene expression in the brain.

NFκB is known to be induced acutely in response to ischemic injury; however, investigation into the role of NFκB activity has revealed conflicting results [2]. For instance, NFκB is constitutively active in neurons, a requirement for their survival, while the surrounding glial cells have inducible NFκB activity [32]. In response to ischemic challenge, NFκB activity in astrocytes is responsible for detrimental inflammation [33]. This concept of pleotropic roles also applies to many of the inflammatory genes expressed in the brain in the setting of stroke [34,35]. For example, intracerebroventricular injection of recombinant IL6 significantly decreased the infarct size in rats 24 hr post MCAO [36]. IL1β is a potent inducer of IL1 receptor antagonist (IL-rn), which significantly reduces damage in response to stroke [37] and, notably, is upregulated in our microarray (Figure 1, Lt.). TNFα is considered to play multiple roles in stroke injury mediating many neuroprotective and injurious effects [34]. Furthermore, in response to viral challenge, the simultaneous presence of inflammatory cytokines, such as TNFα, and type I IFNs can alter their effects and synergize to promote a more protective state [38]. Thus, alterations in the environment in which NFκB is activated and inflammatory genes are present may affect the roles pro-inflammatory mediators play in injury and may even contribute to the protective phenotype.

IRF3 activity induces the expression of anti-inflammatory and type I IFN-associated genes. Interestingly, mice deficient in IRF3 are not protected against cerebral ischemia by LPS preconditioning [13]. We have further established the importance of IRF3 in neuroprotection by identifying that multiple preconditioning paradigms including LPS, CpG (TLR9 agonist) and brief ischemia induce a common set of IRF-mediated genes in the neuroprotective environment following MCAO [14]. Here we demonstrate that IRF3 activity is upregulated in the brain of LPS-preconditioned mice in response to MCAO and that several IRF3-mediated genes are also upregulated, including RANTES and IFIT1 (diagrammed in Figure 7), which may mitigate the damaging effects of ischemia.

Many of the upregulated anti-inflammatory/type I IFN genes in the brain following stroke have several identified neuroprotective functions. TGFβ has been shown to protect neurons from apoptosis, promote angiogenesis, decrease microglial activation, and reduce edema [34,39]. RANTES, which is induced by IRF3 and IRF7 [30], has been shown to protect neurons from cell death in response to HIV-1 glycoprotein gp120 [40]. In the setting of brain ischemia, mice deficient in the RANTES receptor, CCR5, have larger infarcts, suggesting a neuroprotective role for CCR5 activation [41]. Notably, the expression of CCR5 is upregulated in our microarray data (Figure 1, Lt). IFIT1 is commonly associated with IRF3 signaling in response to IFN treatment and viral infection [42]. Little is known about a role for IFIT1 in ischemic injury; however, it is inducible in microglia and neurons and has been shown to affect NFκB and IRF3 activation [42-45]. Additional anti-inflammatory/type I IFN genes shown to be upregulated in our microarray studies have potential roles in neuroprotection including IL-receptor antagonist (IL-rn), which is associated with reduced infarct size in response to stroke [34,46]. A recombinant form of IL-rn is being tested in Phase II clinical trials as an acute stroke therapy [47,48]. Although not detected in our gene microarray studies here, perhaps due to assay sensitivity for IFNβ transcript on the microarray, we have previously published that IFNβ mRNA, a type I IFN known to have neuroprotective properties, is upregulated following stroke in the brain of LPS-preconditioned mice using qtPCR [13]. The protective functions of these genes may be of considerable importance to the neuroprotective phenotype following MCAO induced by LPS preconditioning.

Research strongly suggests that cerebral ischemia dramatically alters the protein and gene expression profile in the peripheral blood [5,29,49,50]. Our results demonstrate that the cytokine and chemokine response in the blood paralleled the pattern of gene expression in the brain. Overall, inflammatory cytokine protein levels were similarly induced in LPS and saline preconditioned mice following stroke. However, we have previously published that TNFα is significantly reduced in the plasma of LPS-preconditioned mice following MCAO [51]. The anti-inflammatory and type I IFN-induced cytokines and chemokines measured in the blood were enhanced in LPS-preconditioned mice compared to saline. In particular, IL10 was significantly upregulated in the blood following MCAO of LPS-preconditioned mice. Importantly, in humans, upregulation of IL10 in the blood has been correlated with improved outcome in stroke [52]. While IL10 mRNA was not detectable in the brain, IL10 can be induced by IRF3 activity and therefore is indicative of the same redirected response seen in the brain. IFNβ was not detected in the blood 24 hr post MCAO. This may be due to the kinetics of IFNβ expression. Further investigation into the time course of IFNβ induction in the blood is necessary to fully understand the role of IFNβ in this system. The redirected signaling observed in the blood may stem from the brain's response to injury by leaking proteins into the peripheral circulation; however, this is not considered a major source of plasma cytokines at these early timepoints following stroke [29]. Alternately, because LPS administration occurs by a systemic route, target cells in the periphery may become tolerant to activation by the secondary stimuli resulting from ischemic injury. Although our data does not distinguish between these possibilities, it is clear that LPS preconditioning alters the response to injury in the brain and the blood in a manner that promotes a protective phenotype.

TLR4 signals through the adaptor molecules MyD88 and TRIF. MyD88 signaling culminates in NFκB activation. TRIF signaling can activate both IRF3 and NFκB, although IRF3 activation often is more rapid and robust, while activation of NFκB is a secondary effect that occurs as part of late-phase TLR signaling [53]. The data presented in this paper and Marsh et al., 2009 [13] suggests a dominant role for IRF3 signaling in LPS-induced neuroprotection, which implicates the TRIF adaptor as a key player in the reprogrammed TLR4 response to stroke. Support for this lies in our finding that mice deficient in TRIF are not protected by LPS preconditioning. In contrast, MyD88 deficient mice preconditioned with LPS are still protected against MCAO. Taken together, these data strongly support a protective role for TRIF-mediated IRF3 activation in the neuroprotective phenotype induced by LPS preconditioning.

TLRs have the ability to self regulate in a manner that redirects their signaling. The classic example is endotoxin tolerance, whereby a low dose of the TLR4 ligand LPS reprograms TLR4 signaling in response to a subsequent toxic dose of LPS, leading to a protective phenotype [54]. This reprogrammed response comes in two major forms: (1.) suppressed pro-inflammatory signaling and enhanced anti-inflammatory/type I IFN signaling, or (2.) enhanced anti-inflammatory/type I IFN signaling in the absence of suppressed pro-inflammatory signaling. Thus, the suppressed NFκB activity, the enhanced IRF3 activity, and the upregulated anti-inflammatory/type I IFN associated genes seen in the LPS-preconditioned brain following stroke is reminiscent of endotoxin tolerance--a phenomenon that has been best described in macrophages *in vitro*, but more recently in animals. Many other key features of endotoxin tolerance are seen in the reprogrammed response to stroke produced by LPS preconditioning. For example, Tollip and Ship1 are known to be induced in endotoxin tolerance and lead to suppressed NFκB activity. TGFβ has been shown to play an important role in endotoxin tolerance, whereby TGFβ-mediated induction of SMAD4 is required to promote complete endotoxin tolerance and to induce the NFκB inhibitor, Ship1 [55]. Interestingly, in our system the upregulation of TGFβ corresponds to Ship1 upregulation 24 hr post MCAO in LPS-preconditioned mice compared to saline. Furthermore, cells deficient in TRIF or IRF3 are unable to develop tolerance to endotoxin [56]. This is similar to TRIF deficient or IRF3 deficient mice not being protected by LPS preconditioning against cerebral ischemia. Taken together, this suggests that the cellular phenomenon of endotoxin tolerance is potentially the same response observed in LPS preconditioning wherein LPS exposure leads to a reprogrammed TLR signaling response in the brain following stroke to produce protection.

## Conclusions

The findings reported here provide an important characterization of the LPS-induced neuroprotective response following stroke. We show that LPS preconditioning induces a reprogrammed response to stroke, whereby NFκB activity is suppressed, IRF3 activity is enhanced, and anti-inflammatory/type-I IFN genes are upregulated (diagrammed in Figure 7). Interestingly, the suppression of pro-inflammatory genes is not a necessary part of the neuroprotective response induced by LPS preconditioning. Further evaluation into the TLR4 signaling cascades revealed a seminal role for the TRIF cascade in producing the neuroprotection initiated by LPS preconditioning. As TRIF signaling culminates in IRF3 activation, this finding provides further evidence for the importance of IRF3 in the neuroprotective response to stroke.

## Competing interests

The authors declare that they have no competing interests.

## Authors' contributions

KBV performed experiments, collected data, conceived of the idea for the paper, and wrote the manuscript. SLS worked on the microarray, provided guidance in the production of data, and edited the paper. BJM performed experiments and contributed to the writing of the Methods section. RWK performed experiments. NL performed the MCAO surgeries. MSP provided critical guidance and worked on the manuscript. All authors approved of the final manuscript.
